# The Impact of Continuity of Care on Health Indicators in Patients With Type 2 Diabetes Mellitus in Family Medicine Clinics in Riyadh

**DOI:** 10.7759/cureus.43410

**Published:** 2023-08-13

**Authors:** Ghada Hussein, Aljoharah A Al Saud, Ahmad M Siddiqi, Abdallah Khasawinah, Ahmad Alenezi, Riham A Mohammed, Yaser A Alendijani

**Affiliations:** 1 Family Medicine and Polyclinics, King Faisal Specialist Hospital and Research Centre, Riyadh, SAU; 2 Family Medicine and Polyclinics, Alfaisal University College of Medicine, Riyadh, SAU

**Keywords:** family medicine, health indicators, diabetes, usual provider continuity score, continuity of care index, continuity of care

## Abstract

Background: Diabetes Mellitus Type 2 (DM2) is highly prevalent in Saudi Arabia, with many experiencing complications due to the disease. Family medicine physicians are usually the primary care providers responsible for the medical management of type 2 diabetes mellitus patients. Microvascular and macrovascular complications can occur if type 2 diabetes mellitus is poorly managed. Effective management of health indicators in patients with DM2 relating to glycated hemoglobin (HbA1c), low density lipoprotein cholesterol, blood pressure, and tobacco use is an essential part of medical care to prevent complications. Due to the projected increase in the number of patients with DM2, there is huge concern surrounding the management of this chronic illness that requires review. This study aims to evaluate the impact of continuity of care on health indicators among family medicine patients diagnosed with diabetes mellitus type 2 and to analyze the effect of continuity of care regarding the completion of age-appropriate preventive health screenings.

Methods: This is a retrospective cohort study. Data collected from electronic medical records of patients 40-75 years of age that received care at the Family Medicine clinics that were diagnosed with type 2 diabetes mellitus with ≥4 clinic visits from January 1, 2017, to June 30, 2020, at King Faisal Specialist Hospital & Research Centre in Riyadh, Saudi Arabia. Data collected included demographic data, body mass index, smoking status, blood pressure, past medical history, preventive health screening completed, and laboratory results, including HbA1c and lipid profile. The continuity of care index and usual provider continuity score indices were calculated for the analysis to measure continuity of care.

Results: Three hundred and fifty-two patients were included in the study. Most of the patients were Saudi (74.15%), female (51.99%), and married (82.67%). In addition, 90.34% accounted for a high usual provider continuity of care score (UPCS), and 64.20% of the patients had a high continuity of care index (COCi). Younger age groups were significantly more prevalent in the high UPCS group (p=0.037). Additionally, patients of non-Saudi nationalities constituted a significantly larger proportion of the high UPCS group. Single patients showed high UPCS. Comorbidities were not different between the groups, except inflammatory joint disease, which was more common in the low COCi group. Preventative screening measures were also not different between the groups; however, the type of colon cancer screening differed, where patients with high COCi more frequently underwent colonoscopies (13.3% vs. 4.4%, p=0.015) instead of fecal occult blood tests.

Conclusion: For the first time, we report the implications of the continuity of care for DM2 patients in Saudi Arabia and the Middle East. Continuity of care did not result in the improvement of health indicators or in the completion of preventive health screenings in diabetic patients. Further studies are needed in the region to confirm our findings and assess the association between continuity of care and patient health indicators impact.

## Introduction

One of the core values of family medicine is continuity of care. The concept of continuity of care is referred to as repeat doctor-office visits between one provider and patient [1]. The World Health Organization (WHO) recommends continuity of care for the management of type 2 diabetes mellitus patients [2]. Higher continuity of care among diabetes mellitus patients resulted in little to no improvement in various health indicators but was associated with improved health outcomes [2]. Type 2 diabetes mellitus is a common medical condition affecting many adults worldwide and is a risk factor for developing coronary artery disease leading to death. The prevalence of type 2 diabetes mellitus in the Saudi Arabian general population is 31.6% [3]. Over the next 10 years, the number of people affected by type 2 diabetes mellitus is expected to increase substantially, which is a significant health concern due to the morbidity and mortality associated with the disease.

Type 2 diabetes mellitus affects mainly adults. Glycated hemoglobin is one of the diagnostic screening tests used to diagnose DM2 [4]. The HbA1c is the average blood glucose over the last 2 to 3 months, and patients with an HbA1c ≥ 6.5% are diagnostic of DM2 [5]. A family medicine provider’s goal in managing DM2 is to maintain blood glucose levels within or close to normal ranges [6]. Uncontrolled DM2 can lead to heart, kidney, eye, and or nerve damage, which contributes to a poor quality of life.

Patients with type 2 diabetes mellitus are at increased risk of developing macrovascular and microvascular complications [4]. The complications can be prevented when patients adhere to the treatment and medication regimen prescribed, follow up with a medical provider regularly, and have diabetes monitored through the use of periodic tests as recommended by the American Diabetes Association [7]. Continuity of care was strongly associated with reduced complication risk in patients with DM2 [2]. Previous studies on health outcomes in newly diagnosed DM2 patients indicated that continuity of care was associated with fewer hospitalizations, fewer emergency room visits, and a lower probability of dementia [2]. 

As the prevalence of DM2 patients increases, this burdens the health care delivery systems, which leads to challenges in providing efficient care to DM2 patients [8]. Most studies have investigated the effect of continuity of care on healthcare outcomes instead of health indicators; therefore, our study will investigate this important effect. To our knowledge, the effects of continuity of care on health indicators have not been investigated in Saudi Arabia before. Thus, with the growing prevalence of DM2 in Saudi Arabia, it is highly important to evaluate the effect of continuity of care as a means of providing ideal medical management to DM2 patients.

## Materials and methods

Retrospective review of the Integrated Clinical Information System (ICIS) database of the electronic health records of patients 40-75 years of age with HbA1c ≥ 6.5% (diagnosed with DM2) that were seen in King Faisal Specialist Hospital & Research Centre (KFSH&RC) Family Medicine clinics with ≥4 clinic visits from January 1, 2017, to June 30, 2020. Patients with fewer than three clinic visits, under care by endocrinology, patients with active cancer or dementia, patients prescribed oral corticosteroids, patients prescribed isotretinion, and patients who are pregnant or postpartum will be excluded. 

The data to be collected include demographics, past medical history, diabetic and statin medication prescribed, body mass index (BMI), tobacco use, and lab results, including HbA1c, LDL-C, and urine albumin/creatinine ratio.

Medical records will be reviewed to collect data if patients have completed age-appropriate preventive health screenings such as breast cancer screening by mammogram, retinopathy screening by ophthalmologist, cervical cancer screening, or colon cancer screening by fecal occult blood test or colonoscopy.

To determine if health indicators are effectively managed, the Saudi Diabetes Clinical Practice Guidelines (SDCPG) will be used. It is recommended for most adults to achieve the HbA1c goal of ≤ 7% since multiple trials showed a 50%-60% reduction in microvascular complications and 57% fewer cardiovascular disease events [9]. Patients with extensive comorbid conditions, limited life expectancy, or advanced complications are recommended to achieve a less stringent target of HbA1C ≤ 8.5% [9]. A blood pressure of ≤ 130/80 is an appropriate target, which has been shown to be beneficial in reducing both micro- and macrovascular complications if it can be achieved safely [9]. The use of statins is associated with lower cardiovascular events in primary and secondary prevention of atherosclerotic cardiovascular disease. A chart review will be performed to determine if statins were prescribed [9]. Smoking cessation is recommended as a routine care component for patients with DM2. A chart review will be performed if this was discussed, if the patient quit smoking, or if medications for smoking cessation were prescribed during the duration of the study [9].

Currently, patients can see any family medicine physician for medical care within KFSH&RC, but for the purpose of this study, the primary physician will be noted as the physician the patient saw the most from their total visits, adopting the continuity of care index. Continuity of Care Index (COCi) is a calculated value; the index scores are between 0 and 1. This reflects a physician’s share of a patient’s total visits, where a higher score (score of 1) is indicative that a patient visited the same physician more [8]. A COCi of 1 is suggestive that the patient consults the same physician, therefore receiving continuity of care, while a score closer to 0 suggests they have been treated by multiple physicians [2,10]. Continuity scores in the upper 50% are defined as high continuity, while those in the lower 50% are defined as low continuity group [2]. The density of care is obtained by the usual provider continuity score (UPCS), the most visited physician is determined, and the result is the proportion of visits with that physician [8]. Studies have determined that a low number of patient visits makes it difficult to determine continuity of care; therefore, the recommendation is to restrict analysis to patients with more than three clinic visits [8].

IBM Corp. Released 2011. IBM SPSS Statistics for Windows, Version 20.0. Armonk, NY: IBM Corp. was used to perform the statistical analysis. Descriptive statistics for the continuous variables were summarized as mean and standard deviation, while the categorical variables were reported as counts and percentages. For inferential statistics, continuous variables such as the laboratory parameters and the change difference were compared using the independent t-test. Categorical variables were compared using the Chi-square test and Fisher's exact test. The significance level was set at 0.05 with a 95% confidence interval. 

Ethical approval was obtained from the Research Ethics Committee at King Faisal Specialist Hospital and Research Center on 24 February 2022 (RAC 2221035), and a waiver of informed consent was granted.

## Results

Three hundred and fifty-two patients were included in the present study, with a mean age of 57.59 ± 8.47 years old. The majority of the recruited patients were female (52%), Saudi (74.1%), married (82.7%), and reported not using tobacco (91.2%). Detailed patient demographics can be found in Table 1. In addition, the mean usual provider continuity score of the study sample was 0.77 ± 0.21, and 90.34% accounted for a high UPCS. The mean continuity of care index was 0.63 ± 0.31, and 64.2% of the patients had a high COCi. The mean body mass index, HbA1c, and low-density lipoprotein cholesterol were 32.13 ± 6.62, 7.89 ± 1.67, and 2.8 ± 0.9, respectively. 

**Table 1:** Demographics of the patient population.

Parameter	Frequency (%)
Gender	
Male	169 (48%)
Female	183 (52%)
Nationality	
Saudi	261 (74.1%)
Non-Saudi	91 (25.9%)
Marital status	
Married	291 (82.7%)
Single	40 (11.4%)
Divorced	5 (1.4%)
Widowed	16 (4.5%)
Tobacco use	
Yes	29 (8.2%)
No	321 (91.2%)
Quit during study period	2 (0.6%)

**Figure 1:**
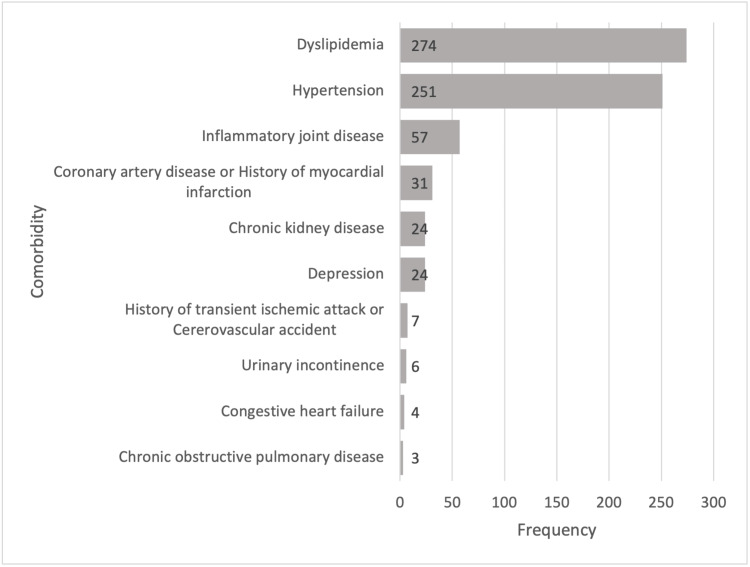
Frequency of patient comorbidities.

Figure 1 highlights the frequency of comorbidities in the cohort population. Dyslipidemia and hypertension were the most common comorbid medical conditions.

Comorbidities were not different between the groups, but there was a statistical significance in patients with inflammatory joint disease, which was more common in the low UPCS (29.4% vs. 14.9%, p=0.047) and low COCi groups (22.6% vs. 12.9%, p=0.019) (Table 2). Furthermore, body mass index, glycated hemoglobin, low density lipoprotein cholesterol, systolic blood pressure, and diastolic blood pressure changes were similar between both groups (Table 3). 

**Table 2:** The association between patient comorbidities and UPCS and COCi. UPCS: Usual provider continuity score, COCi: Continuity of care index

Comorbidities	UPCS		P-value	COCi		P-value
	Low	High		Low	High	
Hypertension	24 (70.6)	227 (71.6)	0.900	93 (73.8)	158 (70.2)	0.475
Dyslipidemia	27 (79.4)	247 (77.9)	0.841	99 (78.6)	175 (77.8)	0.863
Depression	5 (14.7)	19 (6)	0.070	9 (7.2)	15 (6.7)	0.850
Urinary incontinence	2 (5.9)	4 (1.3)	0.107	4 (3.2)	2 (0.9)	0.192
Inflammatory joint disease	10 (29.4)	47 (14.9)	0.047	28 (22.6)	29 (12.9)	0.019
Coronary artery disease (CAD) or History of Myocardial infarction (MI)	4 (11.8)	27 (8.5)	0.523	13 (10.3)	18 (8)	0.463
Congestive heart failure (CHF)	0 (0)	4 (1.3)	0.999	1 (0.8)	3 (1.3)	0.999
History of transient ischemic attack (TIA) or Cerebrovascular accident (CVA)	1 (2.9)	6 (1.9)	0.516	3 (2.4)	4 (1.8)	0.705
Chronic obstructive pulmonary disease (COPD) or Emphysema	0 (0)	3 (1)	0.999	0 (0)	3 (1.3)	0.556
Chronic kidney disease (CKD)	1 (2.9)	23 (7.3)	0.491	9 (7.2)	15 (6.7)	0.867

**Table 3:** Preventive care screenings and their association with UPCS and COCi. UPCS: usual provider continuity score, COCi: continuity of care index, FOBT: fecal occult blood test

Preventive Care Screenings	UPCS		P-value	COCi		P-value
	Low	High		Low	High	
Cervical Cancer			0.531			0.848
Yes	12 (57.1)	74 (45.7)		41 (48.8)	45 (45.5)	
No	6 (28.6)	38 (23.5)		18 (21.4)	26 (26.3)	
Not applicable	1 (4.8)	22 (13.6)		10 (11.9)	13 (13.1)	
Refused	2 (9.5)	28 (17.3)		15 (17.9)	15 (15.2)	
Breast Cancer			0.550			0.117
Yes	11 (52.4)	103 (63.6)		53 (63.1)	61 (61.6)	
No	3 (14.3)	24 (14.8)		9 (10.7)	18 (18.2)	
Not applicable	0 (0)	3 (1.9)		0 (0)	3 (3)	
Refused	7 (33.3)	32 (19.8)		22 (26.2)	17 (17.2)	
Colon Cancer			0.899			0.210
Yes	22 (64.7)	182 (57.2)		78 (61.9)	126 (55.8)	
No	7 (20.6)	68 (21.4)		27 (21.4)	48 (21.2)	
Not applicable	1 (2.9)	15 (4.7)		2 (1.6)	14 (6.2)	
Refused	4 (11.8)	53 (16.7)		19 (15.1)	38 (16.8)	
Type of screening			0.340			0.015
FOBT	31 (96.9)	234 (89.3)		109 (95.6)	156 (86.7)	
Colonoscopy	1 (3.1)	28 (10.7)		5 (4.4)	24 (13.3)	
Retinopathy			0.074			0.694
Yes	22 (64.7)	138 (43.4)		61 (48.4)	99 (43.8)	
No	8 (23.5)	123 (38.8)		45 (35.7)	86 (38.1)	
Refused	4 (11.8)	57 (17.9)		20 (15.9)	41 (18.1)	
Nephropathy			0.030			0.260
Yes	26 (76.5)	236 (74.2)		98 (77.8)	164 (72.6)	
No	8 (23.5)	44 (13.8)		19 (15.1)	33 (14.6)	
Not applicable	0 (0)	38 (11.9)		9 (7.1)	29 (12.8)	

Screening for cervical cancer, breast cancer, colon cancer, retinopathy, and nephropathy was performed more frequently in the high UPCS and COCi groups, but there was no statistical significance between the groups; however, the type of colon cancer screening differed, where patients with high COCi more frequently underwent colonoscopies (13.3% vs. 4.4%, p=0.015) instead of fecal occult blood tests (Table 4). 

**Table 4:** The association between various patient variables and UPCS and COCi. UPCS: usual provider continuity care score, COCi: continuity of care index

Variables	UPCS		P-value	COCi		P-value
	Low	High		Low	High	
Age	58.24 ± 6.79	57.52 ± 8.64	0.641	58.15 ± 8.16	57.28 ± 8.64	0.355
< 45	1 (2.9)	25 (7.9)	0.037	7 (5.6)	19 (8.4)	0.714
45 - 49	3 (8.8)	31 (9.7)		11 (8.7)	23 (10.2)	
50 - 54	3 (8.8)	68 (21.4)		24 (19)	47 (20.8)	
55 - 59	10 (29.4)	76 (23.9)		31 (24.6)	55 (24.3)	
60 - 64	10 (29.4)	44 (13.8)		22 (17.5)	32 (14.2)	
65 - 69	6 (17.6)	31 (9.7)		17 (13.5)	20 (8.8)	
≥ 70	1 (2.9)	43 (13.5)		14 (11.1)	30 (13.3)	
Gender			0.230			0.001
Male	13 (38.2)	156 (49.1)		42 (33.3)	127 (56.2)	
Female	21 (61.8)	162 (50.9)		84 (66.7)	99 (43.8)	
Nationality			0.001			0.003
Saudi	33 (97.1)	228 (71.7)		105 (83.3)	156 (69)	
Non-Saudi	1 (2.9)	90 (28.3)		21 (16.7)	70 (31)	
Marital status			0.046			0.012
Single	0 (0)	40 (12.6)		7 (5.6)	33 (14.6)	
Married	31 (91.2)	260 (81.8)		107 (84.9)	184 (81.4)	
Divorced	1 (2.9)	4 (1.3)		2 (1.6)	3 (1.3)	
Widowed	2 (5.9)	14 (4.4)		10 (7.9)	6 (2.7)	
Tobacco use			0.137			0.324
Yes	1 (3)	28 (8.8)		7 (5.6)	22 (9.8)	
No	32 (97)	289 (91.2)		118 (94.4)	203 (90.2)	

In regard to continuity of care, age (p=0.355) and age groups (p=0.714) were not different between patients with high and low COCi (Table 5). In the high COCi group, males comprised most patients (56.2%), while females comprised the majority of patients (66.7%) in the low COCi group (p=0.001). Additionally, non-Saudis were more common in the high COCi group compared to the low group (31% vs. 16.7%, p=0.003). Single patients were also more prevalent in the high versus low COCi group (14.6% vs. 5.6%, p=0.012). Age groups (p=0.037) were statistically significant in the UPCS group, with 13.5% of patients aged 70 and older receiving a high density of care. Tobacco usage was not different between the two groups (9.8% vs. 5.6%, p=0.324).

**Table 5:** Parameter changes and their association with UPCS and COCi. UPCS: usual provider continuity score, COCi: continuity of care index, BMI: body mass index, HbA1c: glycated hemoglobin, LDL-C: low density lipoprotein cholesterol, SBP: systolic blood pressure, DBP: diastolic blood pressure

Parameters Changes	UPCS		P-value	COCi		P-value
	Low	High		Low	High	
BMI percentage	-3.06 ± 5.66	-1.82 ± 8.10	0.386	-1.70 ± 7.85	-2.07 ± 7.94	0.673
HbA1c percentage	-4.01 ± 16.22	-1.04 ± 16.83	0.327	-2.70 ± 14.23	-0.56 ± 18.02	0.251
LDL-C percentage	0.53 ± 30.14	-1.48 ± 35.28	0.750	-5.32 ± 27.66	1.02 ± 38.12	0.103
SBP percentage	-1.23 ± 9.32	-0.63 ± 7.50	0.667	-0.66 ± 8.23	-0.70 ± 7.38	0.963
DBP percentage	0.05 ± 7.94	0.47 ± 10.03	0.814	-0.48 ± 7.88	0.93 ± 10.76	0.197

## Discussion

In this study, we analyzed the impact of continuity of care on various health indicators in patients with diabetes mellitus type 2. To the best of our knowledge, this is the first study reporting continuity of care in Saudi Arabia and its implications. Several studies have investigated the effect of continuity of care on DM2 and health outcomes in other countries, such as the United States, Taiwan, South Korea, Canada, Australia, Malaysia, the United Kingdom, Portugal, the Netherlands, Finland, Chile, and Israel [2]. Beneficial effects of continuity of care relating to service utilization, mortality, and disease-related complications were reviewed more than health indicators [2]. Similar to other studies, hardly any impact on various health indicators was noted in diabetic patients [2].

Contrary to previous studies, younger patients were more prevalent in the high continuity of care groups in the present study. For example, Shin et al. assessed continuity of care and its related factors in Korean diabetic patients [11]. The authors reported that younger patients between the ages of 20 and 39 demonstrated significantly lower continuity of care. The reasons behind our contradicting findings are unclear. Studies in Saudi Arabia have highlighted that younger diabetic patients exhibit greater knowledge of diabetes [12]. Hence, young patients would be more likely to visit their physician more frequently and adhere to care plans, resulting in a higher continuity of care index. Our study also demonstrates that male patients have greater continuity of care. These findings are in line with other reports of diabetic patients globally [11].

We evaluated the impact of continuity of care on health indicators relating to effective control of HbA1c, LDL-C, blood pressure, and tobacco use, but there was no statistical difference found among the groups. A review of multiple studies on the effect of continuity of care on HbA1c reported inconsistencies; six of twelve studies found a positive association between high continuity of care and improved HbA1c, but not with blood pressure or low-lipoprotein cholesterol [2]. However, a study found that having either a specific provider or a usual site of care without a specific provider led to better glycemic control [7]. It was highlighted in a review of studies performed in the Middle East that patients reported a lack of continuity of care as a barrier to good diabetes management [13]. Furthermore, the study revealed patients in Middle East achieved glycemic control when they had one primary care provider [13]. Family medicine providers (i.e., specialized consultants of family medicine with western medical training experience) in our study handle patient care encounters as if they are the primary care provider to ensure management of DM2 and completion of preventive health screenings are addressed at any visit, which may be a reason behind the lack of similar findings in regard to the impact of continuity care on health indicators.

The present study also assessed the relation between COCi and UPCS in relation to preventative screening measures. We found that patients received adequate preventive health screenings, but no statistical significance was noted in regard to continuity of care. Although age-appropriate preventive health screenings for breast cancer, cervical cancer, colon cancer, retinopathy, and nephropathy were completed more in the high continuity of care group. Past studies reported increased continuity of care and lower mortality [1]. Hence, it is likely that patients with greater continuity of care adhere to the recommended screening and care management plans advised by their doctor, which results in better control of their medical conditions. However, further studies are needed to confirm these findings.

Our study faces several limitations. First, this is a single-center study, and, therefore, our findings may not accurately represent diabetic patients in the Middle East or even Saudi Arabia. Second, a small sample size may limit significant study results. Additionally, there is no consensus on the cutoff for high and low COCi or UPCS, which can lead to difficulty when comparing study findings. Overall conclusions might not be greatly impacted, but they may have greater implications for policymaking [2].

## Conclusions

For the first time, we have reported the implications of continuity of care in Saudi Arabia and the Middle East for type 2 diabetic mellitus patients. Increased continuity of care did not result in improved control of health indicators (i.e., glycated hemoglobin, low-density lipoprotein cholesterol, and blood pressure) or the completion of age-appropriate preventive health screenings. Younger, non-Saudi, and single patients attended clinic visits more often. Similarly, male, non-Saudi, and single patients were linked to greater continuity of care. Increased colonoscopies as a method of colon cancer screening rather than fecal occult blood tests were linked to high continuity of care. Further studies are needed in the region to confirm our findings and assess the association between continuity of care and patient health indicators.
